# COPS5 Conferred the Platinum Resistance in Epithelial Ovarian Cancer

**DOI:** 10.3390/cimb44090271

**Published:** 2022-09-01

**Authors:** Hongqin Zhang, Tianqing Yan, Ailing Zhong, Lin Guo, Renquan Lu

**Affiliations:** 1 Department of Clinical Laboratory, Fudan University Shanghai Cancer Center, No. 270, Dong’An Road, Xuhui District, Shanghai 200032, China; 2 Department of Oncology, Shanghai Medical College, Fudan University, Shanghai 200032, China; 3 Qingdao Institute, Fudan University, 699 Jingshatan Road, Qingdao 266500, China

**Keywords:** COP9 signalosome subunit 5, ovarian cancer, platinum resistance

## Abstract

Development of platinum resistance is one of the major causes of epithelial ovarian cancer (EOC) treatment failure. COP9 signalosome subunit 5 (COPS5) was found to take part in the progression of EOC in our previous study. Herein, we aim to uncover the potential utility of COPS5 in EOC chemoresistance. COPS5 levels were analyzed to define clinic pathologic correlates using a matched tissue microarray and online datasets. The effect of COPS5 inhibition by the lentivirus-mediated short hairpin RNA on cell viability, proliferation and migration was accessed in vitro and in vivo. Results showed that COPS5 was upregulated in patients after platinum resistance. Kaplan–Meier survival curves revealed that COPS5 overexpression was correlated with shorter PFS and OS. COPS5 downregulation inhibited the cell proliferation, migration, and reduced the sensitivity of EOC to platinum. Overall, our data indicated that COPS5 inhibition might represent a new therapeutic strategy for overcoming platinum resistance in patients with EOC.

## 1. Introduction

Cancer of the epithelial ovary (EOC) is the most lethal malignancy in women [1]. Platinum resistance directly contributes to the high fatality-to-case ratio of EOC [2]. The improved understanding of platinum resistance may permit the identification of targets for intervention and may improve the prediction of clinical outcomes [3].

COP9 signalosome subunit 5 (COPS5), which was also known as a nuclear exporter, regulates the nuclear-cytoplasm of various cellular signal transduction-related proteins [4,5]. Our previous studies indicated that COPS5 functions as a nuclear exporter and inducer of cytoplasmic degradation of p27 [6], and it promotes tamoxifen resistance in ER alpha-positive breast cancer by degrading NcoR [7]. In addition, we found that the expression of COPS5 was related to the TNM stage and metastasis of EOC. Therefore, we proposed that COPS5 participates in the heterogeneity of ovarian cancers, and in some patients, tumor cells with high COPS5 expression tend to be resistant to platinum and result in the formation of new metastasis.

Here, we found that amplification and overexpression of COPS5 is presented in platinum-resistant tumors using integrated genomic and functional studies. COPS5 inhibition is also sufficient to resensitize platinum-resistant ovarian cancer cells. Furtherly, we characterized the antitumor efficacy of COPS5 knockdown in nude mice with EOC burden, which showed decreased tumor growth and cell proliferation under platinum treatment. The results of our study therefore provide a theoretical basis for targeting a critical protein in platinum-resistant ovarian cancer.

## 2. Materials and Methods

### 2.1. Tissue Microarray and Immunohistochemistry (IHC)

This study was endorsed by the Ethics Committee of Fudan University Shanghai Cancer Center (FUSCC, Certification No. 050432-4-1212B). Ovarian cancer specimens were collected at the time of surgery in a single institution under approved protocol. Twenty pairs of EOC tissues were acquired from 20 patients undergoing resection of the tumor at the Department of Gynecological Oncology, FUSCC (Shanghai, China) from 2011 to 2017. A pair of tissues was required from the same patients undergoing two different surgeries including the primary tumor excision. Tissue microarrays were constructed and each of the samples were collected in triplicate. IHC staining for COPS5 was performed as previously described [8]. The immunostaining intensity of COPS5 in tumor cells was evaluated independently by two senior pathologists.

### 2.2. Cell Culture

The ovarian cancer cell lines A2780 and SKOV3 were bought from ATCC and cultured in RPIM 1640 medium supplemented with 10% FBS. All cell-based experiments were repeated at least three times.

### 2.3. Western Blotting

Cells were lysed on ice using RIPA lysis buffer (Beyotime Biotechnology, Shanghai, China), and then the protein extracts were separated by SDS-PAGE. The primary antibodies used in this study included β-actin (66009-1-Ig, Proteintech, Chicago, IL, USA), COPS5 (6895, CST), CTR1 (20868-1-AP, Proteintech, Chicago, IL, USA), GSTP1 (15902-1-AP, Proteintech, Chicago, IL, USA), GSTT1 (15838-1-AP, Proteintech, Chicago, IL, USA), TOPO1 (sc-271285, Santa Cruz, CA, USA). The secondary antibodies contained HRP-conjugated Goat Anti-Rabbit IgG (SA00001-2, Proteintech, Chicago, IL, USA) and HRP-conjugated Goat Anti-Mouse IgG (SA00001-1, Proteintech, Chicago, IL, USA).

### 2.4. CCK-8 Assay and Evaluation of Drug Interaction

The cell viability was detected using the CCK-8 reagent, which was measured using the following formula: relative viability (experimental absorbance -background absorbance)/(untreated controls absorbance-background absorbance)/100%. The IC50 values of cisplatin and CSN5i-3 were calculated using nonlinear regression by GraphPad software (GraphPad Prism v3.0).

### 2.5. Colony-Formation Assay

Cells were seeded into six-well plates. After 10–14 days of cultivation in a cell incubator, when visible clones appeared, the cell colony was stained with 0.5% crystal violet and photos were taken under bright place.

### 2.6. Assessment of Cell Cycle

A2780 cells with cisplatin resistance were transfected with shRNAs for 24 h. After transfection, cells were collected, washed in ice-cold PBS, fixed with 70% cold ethanol, and stored at 4 ℃ overnight. The next day, cells were washed with ice-cold PBS, resuspended in PI RNase Staining Buffer (BD Biosciences, East Rutherford, NJ, USA), incubated in the dark for 15 min at room temperature, and then analyzed by flow cytometry (Beckman Coulter Inc., Shanghai, China).

### 2.7. Transwell Migration Assays

The cell migration assay was performed using a Transwell chamber (BD FalconTM, San Jose, CA, USA). These chambers were inserted into 24-well cell-culture plates. Cells in 200 μL serum-free culture solution were seeded on the upper chambers. RPMI-1640 medium with 10% FBS was added into the lower chambers to serve as the chemo-attractant. After incubation for 24 h, the uncrossed cells in the upper chambers were removed, while the migrated cells at the lower side of the membranes were fixed with paraformaldehyde (4%) and stained with crystal violet. Pictures were taken at 400× magnification, and cell numbers from five random microscopic fields were counted for statistical analysis.

### 2.8. In Vivo EOC Xenotransplant Experiment

All animal experiments were performed according to the Guidelines for the Care and Use of Experimental Animals and protocols authorized by the Research Ethical Committee of FUSCC. Female BALB/c mice (4–6 weeks old, 16–18 g; *n* = 10) were purchased from Beijing Vital River Laboratory Animal Technology and maintained under pathogen-free conditions. To investigate the effect of COPS5 signaling on the platinum resistance of EOC in vivo, 1 × 10^7^ SKOV3 shcontrol or shCOPS5 cells were injected into the flanks of the mice. To evaluate drug resistance in vivo, xenografted mice with tumor burden were randomly allocated to one of two groups (*n* = 5 per group) to receive intraperitoneal injections of CBP (50 mg/kg) or PBS (control group) twice per week for 12 days. The xenografted tumor size and mouse body weight were measured every two days. The tumor volume was calculated according to the following formula: 0.5 × L × W2, where L is the tumor size at the longest point and W is the tumor size at the widest point. On day 15, the animals were euthanized with an anesthetic overdose and the tumors were resected, weighed, and stored until further analysis.

### 2.9. Hematoxylin-Eosin (HE) Staining

Tumor tissues from mice were fixed in 10% neutral buffered formalin solution, dried, and embedded in paraffin blocks, which were next cut into sections for HE staining.

### 2.10. Kaplan–Meier Plotter Database

The prognostic value of COPS5 in EOC patients treated with platinum was assessed using the online database Kaplan–Meier plotter as previously described. Briefly, the gene name and treatment were first put into the database to obtain Kaplan–Meier survival plots. According to the median expression level, the cases were generally classified into low- and high-expression groups. A log-rank *p*-value, hazard ratio (HR) and 95% confidence interval (CI) were automatically calculated and presented on the webpage (Kaplan–Meier plotter) [9]. A log-rank *p* < 0.05 was considered statistically significant.

### 2.11. Statistical Analysis

Numeric variables were analyzed using the Student’s *t* test for comparing two groups and *ANOVA* for multiple group comparisons. Chi-square and Fisher’s exact test were used to test categorical variables. GraphPad Prism software was used for graphing and statistical analysis. Online ovarian cancer patient data were downloaded and analyzed from the Gene Expression Omnibus (GEO; https://www.ncbi.nlm.nih.gov/geo/, accessed on 20 April 2022) of “GSE15709” [10]. Statistical analyses were performed in R (version 3.0.1; http://www.r-project.org/, accessed on 2 May 2022) software with the package clusterProfiler (Version 3.8.1) [11] and *p* value less than 0.05 was defined statistically significant.

## 3. Results

### 3.1. COPS5 Correlated with Platinum Resistance of EOC and Involved in Its Prognosis

**Figure 1:**
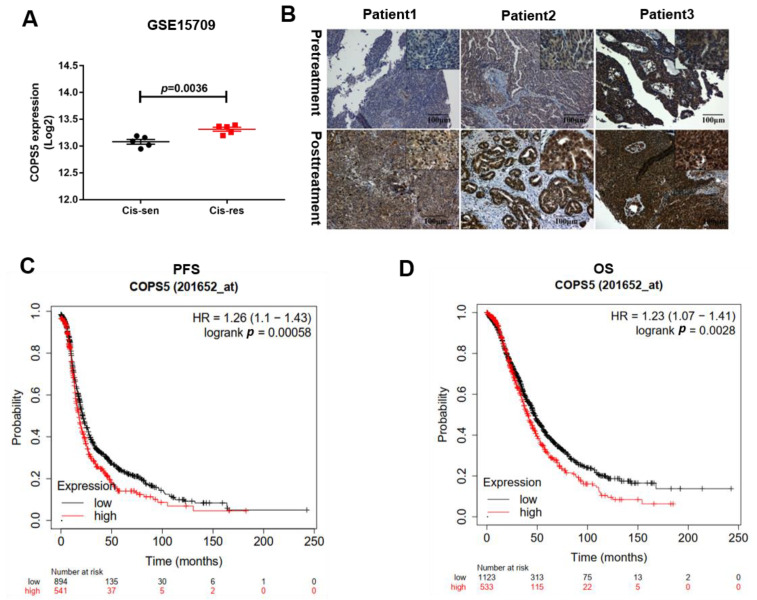
COPS5 is correlated with platinum resistance of EOC and involved in its prognosis. COPS5 was overexpressed in A2780 cisplatin-resistant A2780 cell lines (GSE15709) (**A**). IHC staining was robust in platinum-resistant EOC tissues (**B**). Patients with low levels of COPS5 had a prolonged PFS (**C**). Patients with low levels of COPS5 expression had a prolonged OS (**D**).

In order to understand the potential therapeutic value of COPS5 in platinum-resistant ovarian cancer, we used Gene Expression Omnibus (GEO) datasets to find if there is any relationship between COPS5 and platinum resistance in EOC. GSE15709 is a form of expression and methylation data from A2780 cisplatin-sensitive and A2780 cisplatin-resistant cell lines [10], with both of them having five repeated rounds. We found that COPS5 was overexpressed in A2780 cisplatin-resistance cells (Figure 1A). Next, we performed IHC staining on tissue microarray of 20 patients. Results show that the COPS5 staining in the representative three patients is significantly strong after platinum treatment (Figure 1B). Kaplan–Meier survival curves revealed that COPS5 overexpression was correlated with shorter PFS and OS (Figure 1C,D). The IHC results of twenty pairs of EOC tissues are shown in Figure S1 (see Supplementary Materials). The expression of COPS5 derived from the metastasis tumors on the right were generally higher than the left counterparts. The statistical analysis of the EOC tissue samples according to the intensity of COPS5 staining is exhibited in Figure S2 (*p* = 0.0034). Subsequently, Kaplan–Meier plots were used to assess the prognostic value of COPS5 in EOC. We noticed that of all the patients treated with platinum from TCGA and GEO datasets, those with higher COPS5 expression tend to have shorter PFS. Furthermore, the OS of patients with platinum-treatment and low COPS5 expression seem to prolong than those with COPS5 overexpression (Figure 1D). These results indicated that COPS5 is correlated with the platinum resistance of EOC and involved in its prognosis.

### 3.2. COPS5 Plays an Essential Role in Platinum-Resistant Ovarian Cancer Cell Lines

**Figure 2:**
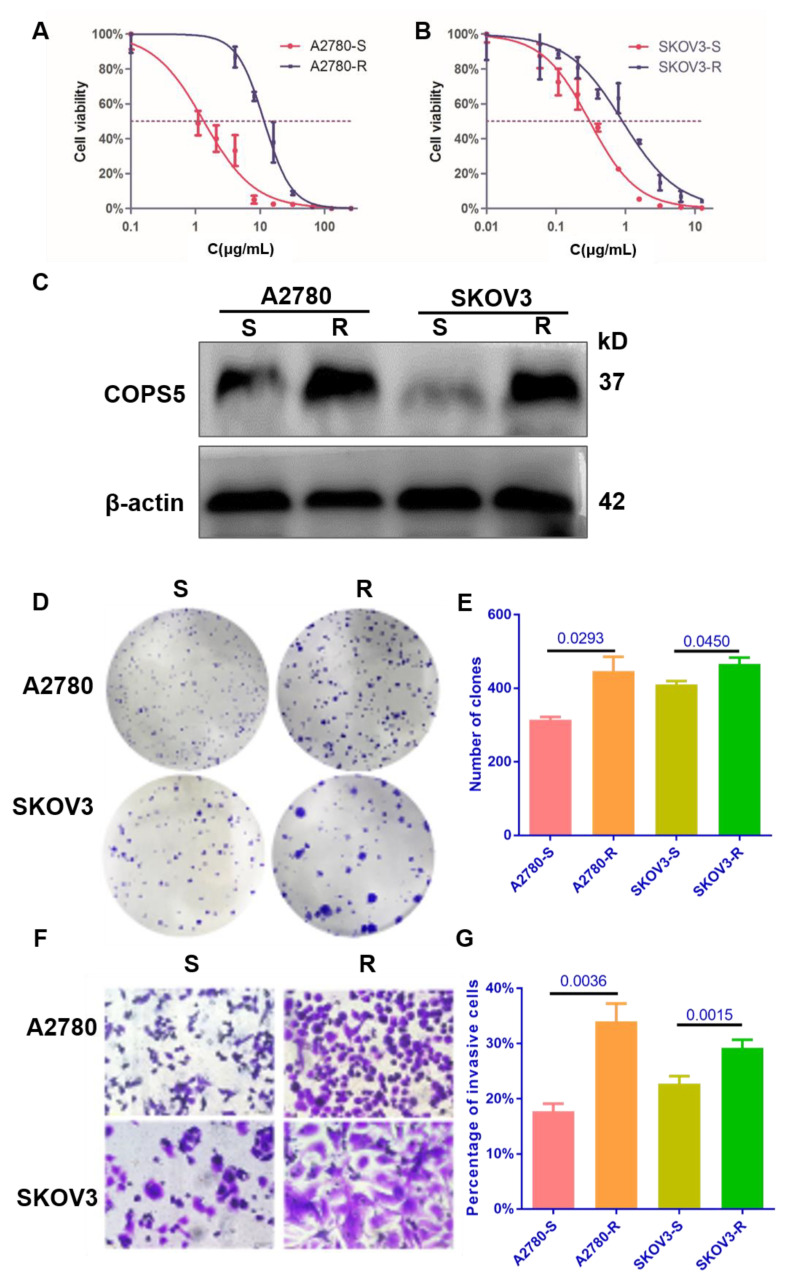
The proliferation and migration of ovarian cancer cell lines were enhanced after platinum resistance. The platinum sensitivity of cell lines (**A**,**B**). The expression of COPS5 in platinum-resistant ovarian cancer cell lines (**C**). The colony formation ability of platinum-resistant cell lines (**D**,**E**). The migration ability of platinum-resistant cell lines (400×) (**F**,**G**). All experiments were carried out in triplicate. A2780-S, platinum-sensitive A2780; A2780-R: platinum-resistant A2780; SKOV3-S, platinum-sensitive SKOV3; SKOV3-R, platinum-resistant SKOV3.

Cell lines resistant to cisplatin have been useful for elucidating the factors that contribute to resistance to platinum. To further verify the expression of COPS5 in platinum-resistant ovarian cancer, we constructed platinum-resistant ovarian cancer cell lines. CCK-8 analysis indicated that the IC50 concentration of the platinum-resistant A2780 cell line was 11.59 ± 0.3584 μg/mL, while the platinum-sensitive A2780 cell line was 1.348 ± 0.247 μg/mL, and the IC50 concentration of the platinum-resistant SKOV3 cell line was 1.326 ± 0.1057 μg/mL, while the platinum-sensitive SKOV3 cell line was 0.3044 ± 0.082 μg/mL (Figure 2A,B). We also found that platinum-resistant related protein CTR1 was downregulated in platinum-resistant A2780 and SKOV3 cells, while GSTP1, GSTT1 and Topo1 were overexpressed (Figure S3), which indicated that the platinum-resistant cell lines were successfully conducted and the expression level of COPS5 in our own constructed cell lines were up-regulated too (Figure 2C). The colonies of platinum-resistant ovarian cancer cells were much larger and greater in number than the platinum-sensitive ones after two weeks of cultivation (Figure 2D,E). Moreover, Transwell analysis showed that platinum-resistant ovarian cancer cells migrated faster than the parental ones. (Figure 2F,G). These results denote that DDP cells demonstrate more aggressive phenotypes, consistent with the clinic phenomenon that ovarian cancer patients with platinum resistance often display tumor regeneration and distant metastasis easier than those who are platinum-sensitive, and with poor prognosis.

### 3.3. Cell Proliferation and Migration Were Inhibited by Down-Regulating COPS5

**Figure 3:**
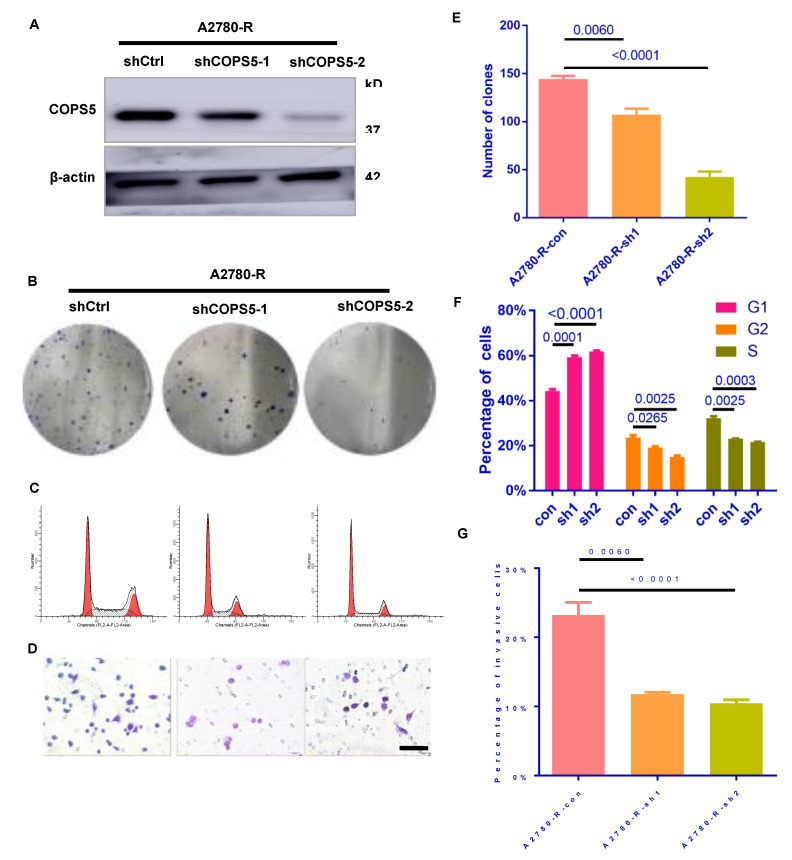
Down-regulation of COPS5 diminished the speed of cell proliferation and migration. Validation of the COPS5 knockdown efficiency (**A**). Effects of COPS5 silencing on the colony formation ability (**B**,**E**). Knockdown of COPS5 in platinum-resistant A2780 cells led to cell-cycle arrest (**C**). The bar graph shows the percentages of each cell cycle phase(**F**). Images of the Transwell assay (400×) and quantification of the Transwell assay (**D**,**G**).

To test the effects of COPS5 on EOC, the lentivirus-mediated RNA interference technique was employed. Western blot analysis confirmed the transfection efficiency (Figure 3A). The colony-forming ability of COPS5 knockdown cell lines was suppressed (Figure 3B,E) and the cell cycle progress was blocked in G1 phase (Figure 3C,F). Transwell analysis revealed that COPS5 silencing inhibited the motility of platinum-resistant A2780 cells (Figure 3D,G).

### 3.4. COPS5 Silencing Sensitized Ovarian Cancer to Platinum In Vivo

**Figure 4:**
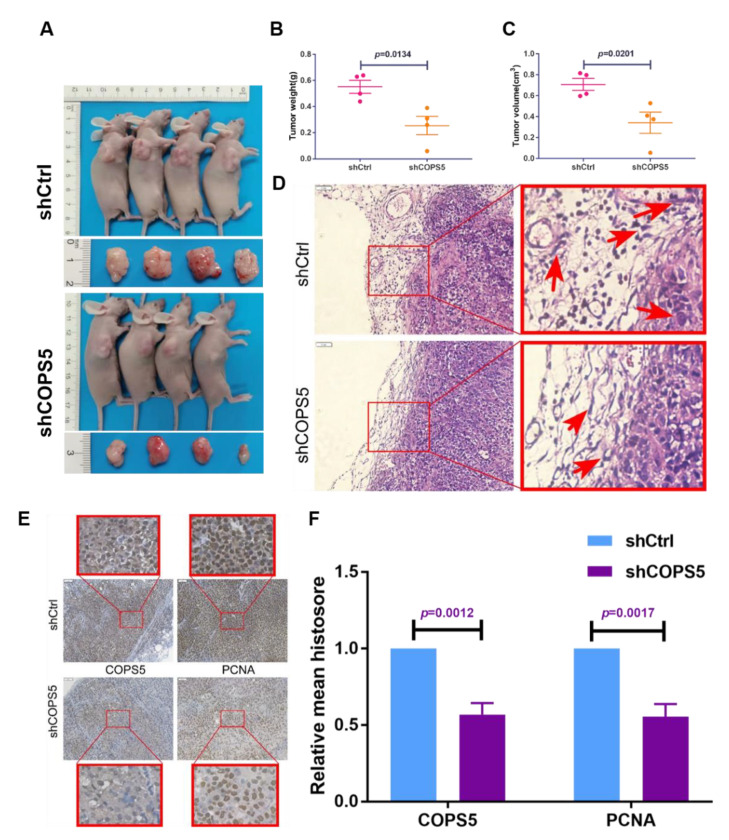
COPS5 silencing sensitized ovarian cancer to platinum in vivo. The tumor growth of mice injected with shCOPS5 ovarian cancer cell line was significantly reduced (**A**–**C**). HE staining showed that the ability of shCOPS5 tumor cells to invade the surrounding tissue was significantly abrogated, 200× (**D**). IHC staining showed that the proliferation ability of shCOPS5 tumor cells was inhibited, 200× (**E**,**F**).

The xenografted mouse model was established to decipher the effect of COPS5 signaling on platinum resistance of ovarian cancer in vivo. Results demonstrated that shCOPS5 significantly diminished the tumor growth in mice (Figure 4A–C). HE staining also demonstrated that the ability of shCOPS5 tumor cells to invade the surrounding tissues was significantly abrogated, 200× (Figure 4D). IHC staining showed that the proliferation ability of shCOPS5 tumor cells was also inhibited, consistent with the experimental results in vitro, 200× (Figure 4E,F).

## 4. Discussion

In this study, we found that COPS5 was correlated with the platinum resistance of EOC and involved in its prognosis. In vitro and in vivo experiments showed that COPS5 knockdown significantly sensitized EOC to platinum, which hinted that COPS5 inhibition is an effective and clinically relevant treatment for platinum-resistant ovarian cancer.

Currently, first-line treatment for ovarian cancer in clinics is debulking surgery followed by platinum-based chemotherapy. Though sensitive to platinum for the first time, more than 70% of patients will be recurrent within 24 months, which is the main cause of failure in EOC treatment [12]. Moreover, due to the genomic heterogeneity within tumors, different molecular and outcome characteristics exist in patients even sharing the same histology [13], which adds greatly to the difficulty of evaluating or interpreting response to therapies and prognoses in patients. Currently, there is no standardized guidance or consensus on how to treat these patients. In this study, data of platinum-interventional A2780 cells from GEO datasets showed that COPS5 were overexpressed in platinum-resistant A2780 cells (GSE15709) [10] (Figure 1A). To further verify the overexpression level of COPS5 in platinum-resistant patients, we gathered 20 pairs of patients’ tissues separated from the surgery before and after platinum treatment and then accessed the expression of COPS5 by immune-histochemical analysis (Figure 1B and Figure S1). We found that COPS5 expression level were increased in platinum-resistant metastasis tissues (*p* < 0.05). In addition, Kaplan–Meier survival curves revealed that COPS5 overexpression was correlated with shorter PFS and OS (Figure 1C,D). These results indicates that COPS5 enzyme contributes to EOC’s platinum resistance and could serve as a prognosis and platinum-resistant related marker for these patients.

To date, the mechanisms of platinum resistance that have been identified are as follows: decreased intracellular concentration due to reduced drug uptake or increased excision of the adducts from DNA repair pathways or alterations of regulatory proteins in signal transduction pathways [14,15,16]. For example, the copper transporter 1 (CTR1) is crucial for platinum uptake into cells, and cells deficient in it are resistant to cisplatin [17,18]. Glutathione (GSH) can cause resistance by binding and inactivating cisplatin, enhancing DNA repair, or reducing oxidative stress caused by cisplatin [19,20]. Glutathione-S-transferase (GST) may augment resistance by catalyzing GSH-drug binding and is also correlated with cisplatin resistance clinically [21]. Resistance to cisplatin due to increased inactivation by intracellular proteins has also been reported [22,23]. Normal cellular function depends on nuclear-cytoplasmic transport [24], and hematologic and solid tumors have increasingly been found to contain abnormalities in this pathway. [22]. Nuclear exportin 1 (XPO1) is one of eight nuclear exporters known to regulate nuclear-cytoplasmic partitioning of tumor suppressor nuclear export sequences (NES). [25], and recent study indicated that XPO1 can also serve as a therapeutic target for platinum-resistant ovarian cancer [26].

Cell lines resistant to cisplatin have been useful for elucidating the factors that contribute to resistance to platinum [21]. In this study, we constructed platinum-resistant EOC cells by prolonged exposure of 5 μg/mL of platinum for at least half a year and then tested the resistance of the cells by CCK-8 and Western blot (Figure 2A–C). In this way, we built an in vitro model for the research of platinum resistance of ovarian cancer. The IC50 in the platinum-resistant A2780 and SKOV3 cells were increased profoundly. In addition, platinum-resistance-related protein CTR1 was downregulated in platinum-resistant A2780 and SKOV3 cells, while GSTP1, GSTT1 and Topo1 were overexpressed, which indicated that the platinum-resistant cell lines are successfully conducted and the expression level of COPS5 in our own constructed cell lines were up-regulated too (Figure 2B and Figure S3). We also found that not only the volume of the platinum-resistant cells was increased, but also the clonal formation and migration ability were enhanced (Figure 2D–G). After suppressing the expression and function of COPS5 by shRNA, we found that the clone formation ability of platinum-resistant cells was suppressed (Figure 3B,E), and suppression of COPS5 would result in G1 phase arrest of A2780 platinum-resistant cells (Figure 3C,F), which was in accordance with our previous finding. COPS5 knockdown also inhibited the migration of platinum-resistant cells (Figure 3D,G). Additionally, in vivo xenografted mouse model results showed that COPS5 silencing significantly diminished the tumor growth (Figure 4). Collectively, the results demonstrated that inhibition of COPS5 was significantly associated with increased tumor killing.

In total, we proposed for the first time that COPS5 was a platinum-resistant protein in both clinical research and in in vitro and in vivo experiments. COPS5 overexpression could define a group of patients with poor prognosis, which provides the rationale for COPS5 as a therapeutic target in platinum-resistant ovarian cancer. However, the full roster of cancer-relevant cargo mediated by COPS5, and the dysregulated pathway caused by inappropriate nuclear-cytoplasmic partition due to upregulation of COPS5, remain unclear and we will continue to unravel the underlying mechanisms in the future.

## Data Availability

Data from this study are available to researchers who obtain permission from the corresponding author.

## Supplementary Materials

The following supporting information can be downloaded at: https://www.mdpi.com/article/10.3390/cimb44090271/s1, Figure S1: The IHC results of twenty pairs of EOC tissues.; Figure S2: The statistical analysis of the EOC tissue samples according to the intensity of COPS5 staining.; Figure S3: The expression of platinum-resistant related protein.
