# Supplementary material for: COPS5 Conferred the Platinum Resistance in Epithelial Ovarian Cancer
Source: Curr Issues Mol Biol. 2022 Sep 1;44(9):3948–58. doi: 10.3390/cimb44090271 (PMC9498275; doi:10.3390/cimb44090271)
Supplement: Supplementary file 1 [file cimb-44-00271-s001.zip › cimb-1862606-supplementary final/supps.pdf]

Supplementary Materials

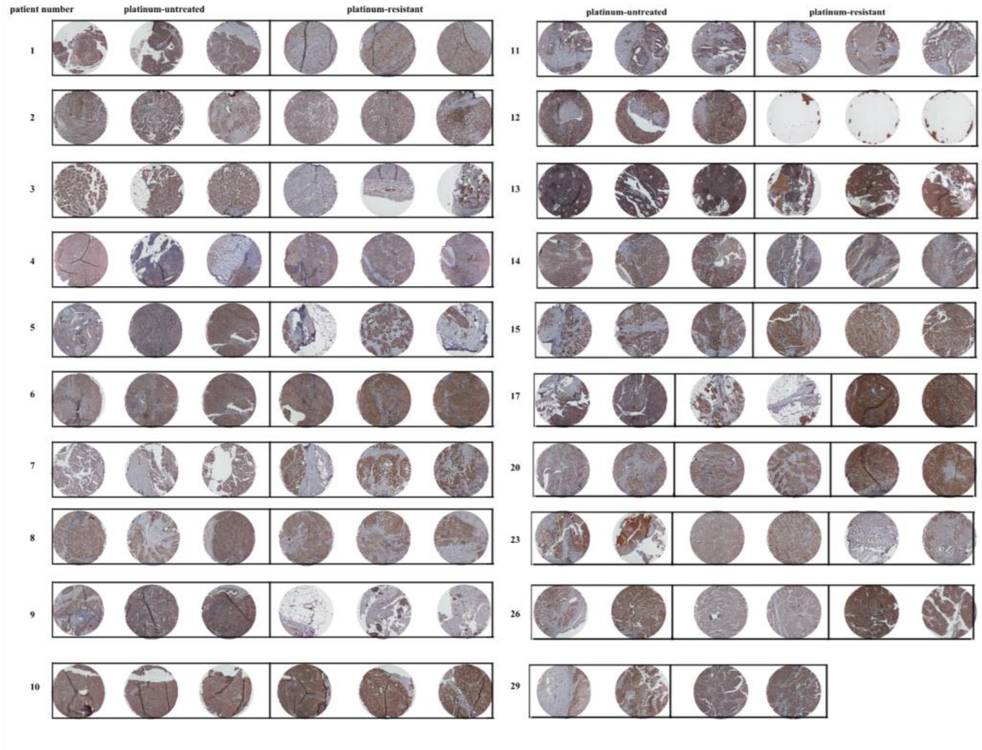

Figure S1. The IHC results of twenty pairs of EOC tissues.

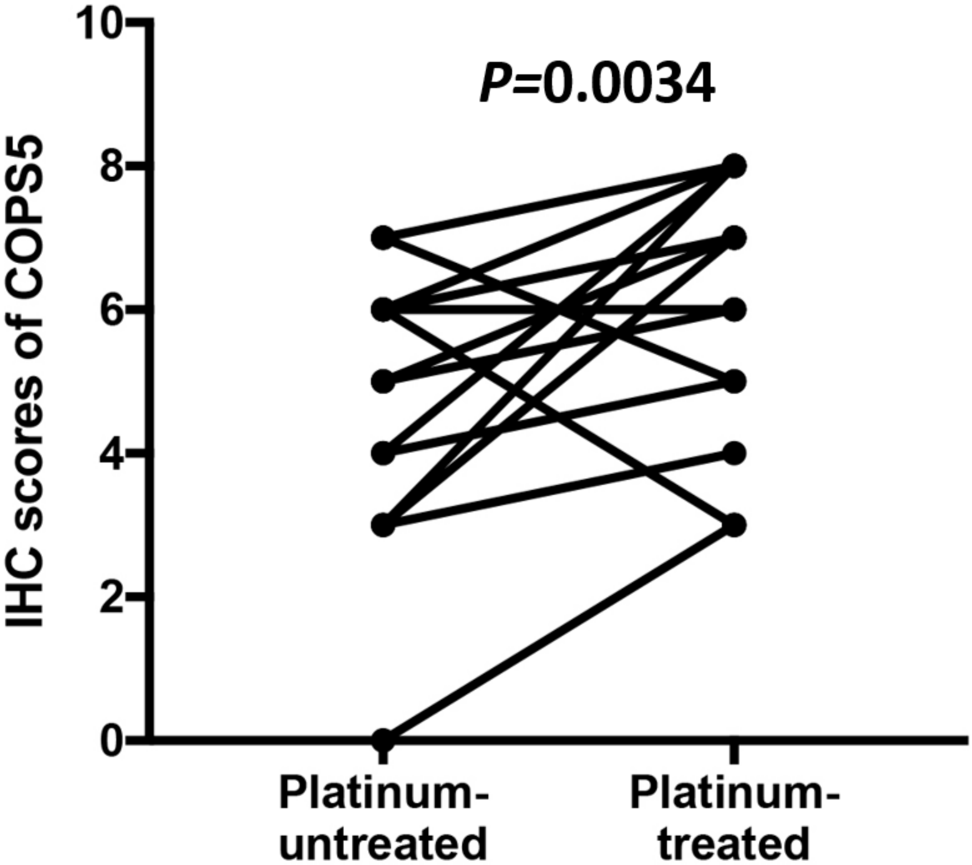

Figure S2. The statistical analysis of the EOC tissue samples according to the intensity of COPS5 staining.

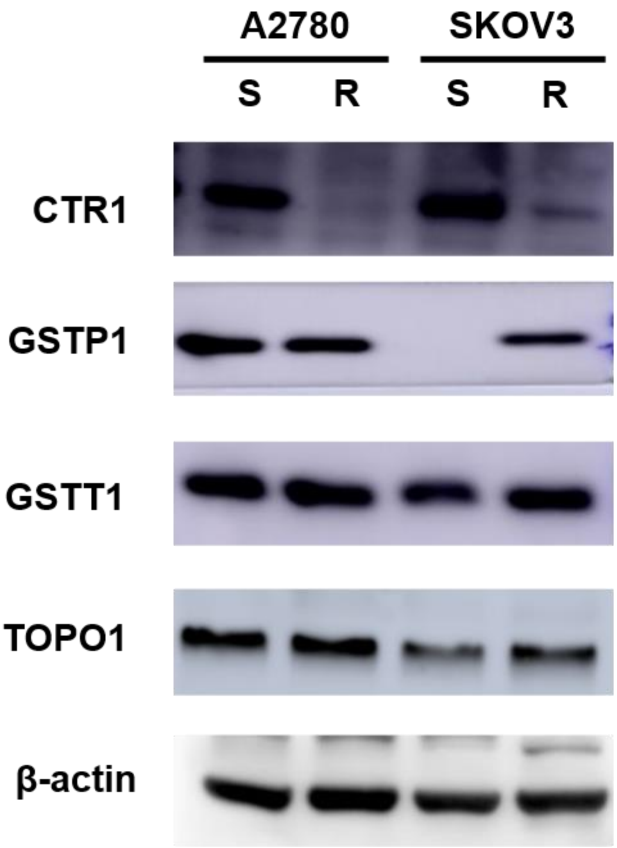

Figure S3. The expression of platinum-resistant related protein.
